# Neuropsychiatry in Demyelination Disease: Using Depression as a Prodrome for Early Diagnosis and Treatment of Multiple Sclerosis

**DOI:** 10.7759/cureus.1813

**Published:** 2017-11-01

**Authors:** Mrigank S Shail

**Affiliations:** 1 School of Medicine, Xavier University School of Medicine

**Keywords:** ms, multiple sclerosis (ms), depression, neuropsychiatry, psychiatry, relapsing remitting multiple sclerosis, myelination, neurology, demyelinating disease, neurons

## Abstract

Multiple sclerosis (MS) is an autoimmune disorder where the body attacks its own insulating myelin sheaths covering the nerve cells in the brain and spinal cord. MS patients show signs of mental illness via emotional blunting, liability, apathy, depression, irritability, and psychosis. Many psychiatrists have noted that the symptomatology of mood disorder is very similar to early signs of MS. The mechanism behind the relationship of depression with MS is not entirely understood at this point. However, through advancements in medical imaging techniques, there are now some leading explanations. One main explanation suggests that depression and memory disturbance are correlated to the demyelination within the limbic system caused by MS. Studies showed that following a diagnosis of MS, the rates of depression are significantly elevated in patients. Several studies noted a lifetime prevalence of major depression in >50% of MS patients. These studies foreshadow that depression is a very important clinical harbinger of active demyelination in MS patients. Depression may hint at which subgroup or stage the MS patient is in, without needing to wait for dramatic physical signs or symptoms to commence. Future physicians may be able to use depression as a prodrome for multiple sclerosis and narrow down the prognosis of their patients, treating them earlier.

## Editorial

It is important to understand and differentiate mood disorders psychopathically from mood disorders caused by a medical condition. From the beginning, it has been clear that multiple sclerosis (MS) patients show signs of mental illness via emotional blunting, liability, apathy, depression, irritability, and psychosis. It is very easy for a psychiatrist to diagnose a patient with depression while missing the diagnosis of MS or without knowing that the signs and symptoms might be a harbinger or complication of MS. At the same time, a neurologist might be treating a patient’s MS, while neglecting the depression without realizing the two might be connected. Many psychiatrists have noted that the symptomatology of mood disorders is very similar to early signs of MS. Therefore, psychiatrists must be careful to not over-diagnose patients with purely depression when, in reality, it might be that depression is a symptom caused by the underlying medical condition of MS.

Multiple sclerosis is an autoimmune disorder where the body attacks its own insulating myelin sheaths covering nerve cells in the brain and spinal cord. The damaged nerves hence cannot communicate properly, resulting in a spectrum of signs and symptoms, ranging from blurred vision to paralysis. The cause of MS is not fully understood yet, but most theories connect the disease to a combination of genetics, environmental factors, and infectious agents. There are four subtypes of MS described in most literature: 1) relapsing and remitting, 2) secondary progressive, 3) primary progressive, and 4) clinically isolated syndrome. The prognosis of MS depends on a degree of factors, which includes the former subtypes. Most individuals with MS reach close to the average life expectancy of an unaffected individual. Among the causes of death in MS patients, infections, paralysis, and suicides are the most common.

In a review of emotional disorders in Multiple Sclerosis, caution should be taken regarding the fact that an overlap of physical symptoms of the disease in terms indexed on personality measures, such as the Minnesota Multiphasic Personality Inventory or the General Health Questionnaire, may lead to inflated estimates of psychopathology in these patients. Hence, it would behoove clinicians to fully grasp the concept of neuropsychiatry in the disease of MS. Physicians must take extra care with MS patients because enough evidence shows that these patients have a higher prevalence of depression than many other neurological patients [1]. When compared to other conditions with a much worse prognosis, such as temporal lobe epilepsy and amyotrophic lateral sclerosis (ALS), MS patients show a significantly higher rate of depression. A University of Rochester study involving groups of epileptic, ALS, and MS patients showed that 62% of MS patients received a depressive spectrum diagnosis - more than that for any of the other diseased groups. This was a significant finding, as it showed that depression in MS could not just be a mental reaction due in part to the physical disability [2].

Even if depression is not seen early in patients with MS, it certainly becomes a significant part of their later life. Feinstein’s, The Clinical Neuropsychiatry of Multiple Sclerosis, presented studies that showed that following a diagnosis of MS, the rates of depression are significantly elevated in patients. Some studies reported a 54% lifetime prevalence of depression, while several studies discovered a lifetime prevalence of major depression in 50% of MS patients [3].

The mechanism behind the relationship of depression with MS is not entirely understood at this point. However, through advancements in medical imaging techniques, there are now some leading explanations. One main explanation suggests that depression and memory disturbance are a consequence of (or at least correlated to) the demyelination within the limbic system caused by MS. Magnetic Resonance Imaging (MRI) studies have found a correlation between measures of memory performance and the total area of lesion involvement.

Using MRI, a five-year Thompson and colleague longitudinal study on changes in the brain makeup of MS patients showed that patients who had the chronic-progressive subgroup of multiple sclerosis were more physically disabled and had developed more extensive brain plaque. These same patients scored three times higher on the depression scale than MS patients with undeveloped plaques and relapsing-remitting MS patients. The interaction between depression, clinical state, and brain lesion load is complex. Changes in brain MRI are not always represented by changes in neurological status and ancillary physical disability. The possibility exists that changes in mood and cognition are more sensitive markers of alterations in brain lesion scores than physical signs [3]. Another 1993 longitudinal Feinstein study showed amplified depression in MS patients with an increased number and size of brain lesions. The study went against the grain because despite the deteriorating MRI picture, there was no concomitant decline in physical disability according to scores on the Expanded Disability Status Scale (EDSS). Patients had become increasingly depressed and their worsening mood scores were significantly correlated with their MRI changes.

What these studies foreshadow is that depression is a very important clinical harbinger of active demyelination in MS patients. Depression may hint at which subgroup or stage the MS patient is in, without needing to wait for dramatic physical signs or symptoms to commence. Some of the newest findings have further narrowed brain regions where lesions are thought to be causing depression. Data now suggest that frontal lobe lesion and temporal lobe atrophy are the predominant factors that distinguish a depressed MS patient from one who is not depressed [4]. Even with these advances in imaging techniques, MRIs lack diagnostic specificity and sensitivity and, therefore, is not the best tool to diagnose depression in MS. MRI methods are not always the gold standard for cerebral pathologies, and (as of now) scans cannot detect brain anomalies in depressed MS patients a majority of the time.

Depression in multiple sclerosis patients is prevalent and can be treated. Several studies promote the use of selective serotonin reuptake inhibitors (SSRI) or selective noradrenaline reuptake inhibitors (SNRI) as a drug of choice [3]. These antidepressants are the same first-line pharmacological medications used for clinically depressed psychiatry patients. In one study, all MS patients who remained on the SSRI sertraline for three months decreased depression without any side effects. The study also noted that prematurely stopping the treatment caused depression to return in 50% of the patients [5]. Apart from treating depression in MS, drugs known as monoclonal antibodies have shown much promise in the treatment of the disease of MS.

Ultimately, most physicians must recognize that depression and MS are intertwined. Understanding the symbiotic relationship of mental or mood changes along with the physical changes of a diseased patient can be highly beneficial. The medical community should use all data gathered to differentiate depression as a mood disorder from depression caused by multiple sclerosis. Looking to the future, physicians may someday be able to use depression as a prodrome - an early sign or symptom - of multiple sclerosis and narrow down the prognosis of their patients, treating them earlier. As always, further research and improved technology are needed to better understand and detect the mechanism behind depression in multiple sclerosis.
